# Development of multiplex real-time PCR for simultaneous detection of SARS-CoV-2, CCoV, and FIPV

**DOI:** 10.3389/fvets.2024.1337690

**Published:** 2024-07-10

**Authors:** Yan Liu, Zhen Zhu, Jige Du, Xiaojie Zhu, Chenfan Pan, Chunsheng Yin, Weidong Sun

**Affiliations:** ^1^Animal Laboratory, China Institute of Veterinary Drug Control, Beijing, China; ^2^College of Veterinary Medicine, Nanjing Agricultural University, Nanjing, China

**Keywords:** real-time PCR, FIPV, canine coronavirus, SARS-CoV-2, detection

## Abstract

**Introduction:**

Coronaviruses, including severe acute respiratory syndrome coronavirus 2 (SARS-CoV-2), canine coronavirus (CCoV), and feline infectious peritonitis virus (FIPV), have the potential for interspecies transmission. These viruses can be present in complex environments where humans, dogs, and cats coexist, posing a significant threat to both human and animal safety.

**Methods and results:**

In this study, we developed a novel multiplex TaqMan-probe-based real-time PCR assay for the simultaneous detection and differentiation of SARS-CoV-2, CCoV, and FIPV. Specific primers and TaqMan fluorescent probes were designed based on the N region of SARS-CoV-2 and FIPV, as well as the S region of CCoV, which demonstrated a remarkable sensitivity and specificity toward the targeted viruses, as few as 21.83, 17.25 and 9.25 copies/μL for SARS-CoV-2, CCoV and FIPV, respectively. The standard curve constructed by the optimized method in our present study showed a high amplification efficiency within or near the optimal range of 91% to 116% and R(2) values were at least 0.95 for the abovementioned coronaviruses. A total of 91 samples, including six plasmid mixed mock samples, four virus fluid mixing simulated samples, and 81 clinical samples, were analyzed using this method. Results demonstrated strong agreement with conventional approaches.

**Discussion:**

By enabling the simultaneous detection of three viruses, this method enhances testing efficiency while decreasing costs. Importantly, it provides a valuable tool for the prevalence and geographical distribution of suspected and co-infected animals, ultimately contributing to the advancement of both animal and public health.

## Introduction

Coronaviruses are giant, single-stranded positive-sense RNA viruses with an envelope, belonging to the order Nidovirales, family Coronaviridae, and genus Coronavirus (1, 2). According to genetic distance and phylogenetic tree, coronaviruses can be classified into four genera: α-coronaviruses, β-coronaviruses, γ-coronaviruses, and δ-coronaviruses (3). α- and β-coronaviruses primarily infect mammals, while γ- and δ-coronaviruses have a tendency to infect birds (3, 4). Among these coronaviruses, CCoV and FIPV are classified as α-coronaviruses, whereas SARS-CoV-2 belongs to β-coronaviruses (5). Coronaviruses have a broad host range, which cause a wide spectrum of diseases in both humans and animals, ranging from mild respiratory, enteric, neurological, and renal diseases to more severe manifestations (6).

Despite the restricted host range of coronaviruses, they demonstrate a significant ability to transmit across species (7). The recent emergence of the SARS-CoV-2 virus serves as compelling evidence of its natural in various domestic and wild animals, thereby adding complexity to its epidemiology (8). Additionally, Shi et al. (9) demonstrated that cats exhibited a highly susceptibility to SARS-CoV-2 infection, while dogs displayed comparatively lower susceptibility.

Cats and dogs, as companion animals, have increased significantly in population recently, leading to frequent interaction with humans and other animals. This presents a potential risk as they can serve as sources and sentinels for a wide range of infectious diseases, potentially facilitating cross-species virus transmission (10). SARS-CoV-2 was initially detected in Wuhan, China, in February 2019 and since then, it has sparked a prolonged and devastating pandemic characterized by acute respiratory syndrome in humans. As of 27 August 2023, there have been over 770 million confirmed cases and 6.9 million fatalities (11–13). SARS-CoV-2 may have originated from a bat coronavirus, sharing 96% genome identity (14). Notably, variant shifts, such as Alpha to Delta and Delta to Omicron, are common during the evolution of SARS-CoV-2 (15). Among the various variants, the majority cause symptoms such as fever and cough, causing lower respiratory tract disease, while only a few cause diarrhea (16, 17). CCoV is closely related to enteric coronaviruses found in cats and pigs (18). It is classified into two genotypes, I and II, with CCoV type I showing genetic similarity to feline coronavirus (FCoV) type I rather than CCoV type II (19). CCoV type II can be further categorized into two subtypes, CCoV-IIa (the classical strain) and CCoV-IIb, with the latter believed to have recombined from CCoV-IIa and transmissible gastroenteritis virus (TGEV) (20). Both genotypes can lead to gastrointestinal infections in dogs, and it is likely that mild symptoms or asymptomatic carriage may result from the infection (21). The first report of FIPV dates back to 1963 by Holzworth, and since then, it has spread widely worldwide with high mortality rates (22). FCoV is divided into feline enteric coronavirus (FECV) and FIPV based on its pathogenicity, and these two biotypes are merely virulence variants of the same virus (23). Each biotype can be further divided into two types, FCoV I and FCoV II, based on their antigenicity, with FCoV II arising from the recombination of FCoV I with CCoV-II (24, 25). Symptoms of FIPV can be categorized as either ‘wet’ or ‘dry’ types, which include fibrous peritonitis, pleurisy, vasculitis, diffuse pyogranuloma, uveitis and other manifestations. The presence of ascites is a prominent characteristic of the ‘wet’ type (26).

So far, SARS-CoV-2, CCoV, and FIPV have caused substantial economic disruptions and human fatalities on a global scale. Furthermore, their capacity to traverse species barriers poses a significant threat to public health. In our study, we present a highly efficient and precise multiplex real-time PCR method for detecting these three viruses.

## Materials and methods

### Primers and probes

Typical sequences of SARS-CoV-2, CCoV, and FIPV were obtained from GenBank and analyzed for optimal primer using the MEGA software. The conserved N region was chosen for designing primers and probes for SARS-CoV-2 and FIPV, while the S region was selected for CCoV. As shown in Table 1, three amplification primers and hydrolysis probes were designed using Beacon Designer 8.0 software. Additionally, three extra primers were designed for plasmid construction. Primers implemented in the real-time PCR were designed to have an approximate annealing temperature of ca 51°C. Probes with an annealing temperature of approximately 61°C for SARS-CoV-2, CCoV and FIPV were labeled with Texas Red, Cy5, and FAM, respectively. Both primers and probes were synthesized by Shanghai Sangon Biotech Co., Ltd. (Shanghai, China).

**Table 1:** Primers and probes.

Pathogens	Primers and probes	Sequences (5′ end to 3′ end)	Length (bp)	Gene	Position
SARS-CoV-2	SA-N-F	GCAGGAAGAAGAGTCACAGT	679	N	28,722–29,400^a^
	SA-N-R	TCTACGCAGAAGGGAGC			
	SA-F	CATTCCGAAGAACGCTGA	165		28,997–29,161^a^
	SA-R	ACTGCCACTAAAGCATACAA			
	SA-P	Texas Red-CCTTGTCTGATTAGTTCCTGG-MGB			
CCoV	CC-S-F	CTAAGTCATTAATTTCACCAGTC	462	S	89–550^b^
	CC-S-R	ATTCTGTGGTAATGGTACACATT			
	CC-F	CATAGTTCTGGGAGTCAAATAG	137		178-314^b^
	CC-R	CTTATGAAACCGTGACAGC			
	CC-P	CY5-ACAGCGTCAACTGGACATCCT-BHQ2			
FIPV	FI-N-F	ATGGCCACACAGGGACAAC	1,134	N	27,014–28,147
	FI-N-R	TTAGTTCGTAACCTCATCAATCATCTCAAC			
	FI-F	AAACACACCTGGAAGAAAAC	134		27,701–27,834^c^
	FI-R	GCTATCTGAGGGTAGCATTT			
	FI-P	FAM-CCATTGGCAACGAGATCACTATC-BHQ1			

^a^Genbank number No. OR587279.1.^b^Genbank number No. KP281589.1.^c^Genbank number No. OQ311323.1.

### Cells and viruses

Crandell Reese Feline Kidney (CRFK) and F81 cells were used to cultivate CCoV and FIPV. Other vaccine virus strains, including SARS-CoV-2, feline panleukopenia virus (FPV), feline herpesvirus (FHV), feline caliciviruses (FCV), canine distemper virus (CDV), canine parvovirus infection (CPV), infectious canine hepatitis virus (ICHV), canine adenovirus type 2 (CAV-2), Canine parainfluenza virus (CPIV), were all provided by the China Institute of Veterinary Drug Control. The viral titers were calculated by the endpoint dilution assay (50% tissue culture infective dose [TCID_50_]) according to the Reed-Muench method.

### RNA extraction and reverse transcription

Viral RNA (CCoV and FIPV) was extracted from 100 μL of supernatant using the viral genomic RNA extraction kit [Tiangen Biochemical Technology (Beijing) Co., Ltd.], following the manufacturer’s instructions. Reverse transcription was performed using the 5 × PrimeScript RT Master Mix (TaKaRa Biotechnology Co., Ltd). The cDNA fragment of SARS-CoV-2 was provided by the China Institute of Veterinary Drug Control. To prevent template degradation, the cDNA underwent proper dilution with nuclease-free water, was separated into smaller volumes for individual use, and then preserved at a temperature of −20°C.

### Construction of plasmid standards

Recombinant plasmids carrying the PCR amplicon of the target viruses were cloned and served as artificial templates for plasmid standards. The standard fragments of the target viruses were amplified separately via PCR using the cDNA obtained in the previous step with the Primestar Mix (TaKaRa Biotechnology Co., Ltd). Further, the purified amplification products were recovered by the Omega gel extract kit according to the instructions. They were then cloned into the pTOPO-Blunt vector (Zero Background pTOPO-Blunt Cloning Kit, Aidlab Biotechnologies Co., Ltd) and transformed into DH5α (Beijing Solaibao Technology Co., Ltd.). Plasmids of positive clones (SA-N, CC-S, FI-N) were extracted with plasmid kit II (Tiangen Biochemical Technology (Beijing) Co., Ltd.), which were confirmed via enzyme analysis and DNA sequencing.

The plasmids were quantified using the NanoDrop One (Thermo Scientific) at 260/280 nm UV absorption, and the copy number was calculated. Subsequently, plasmids were 10-fold serially diluted, ranging from 2.183 × 10^10^ to 2.183 × 10^0^ copies/μL for SARS-CoV-2, 1.725 × 10^10^ to 1.725 × 10^10^ copies/μL for CCoV and 0.925 × 10^10^ to 0.925 × 10^10^ copies/μL for FIPV.

### Singleplex real-time PCR reaction cons

The reaction conditions were optimized using varying the volumes of primer and probe (0.24, 0.36, 0.48, 0.60 μM) and annealing temperatures (56°C, 58°C, 60°C, and 62°C) with 10^7^ copies/μL standard plasmids. The real-time PCR reactions had a total volume of 25 μL, consisting of 12.5 μL of 2 × Probe qPCR Mix (TaKaRa Biotechnology Co., Ltd), primer pair (10 μM), probe (10 μM), 1 μL of template, and the remaining volume of nuclease-free water.

Amplification was carried out on a LightCycler® 480 Instrument II (Roche Life Science) using the following program: 95°C for 30 s followed by 45 cycles for each target gene at 95°C for 10 s and 58°C for 30 s. The annealing temperature was determined during the optimization of the reaction system. At the conclusion of each cycle, the acquisition of fluorescence signals was recorded and analyzed with the LightCycler 480 Software and Launch Software add-on for the LightCycler 480 instrument. Standard curves and equations were prepared using Microsoft Excel 2016 to validate the dependability of the dilution product.

### Multiplex real-time PCR reaction conditions

Three primer pairs, probes and the template of the three mixed standard plasmids were added in the multiplex real-time PCR reactions. Following the aforementioned optimization, the concentrations of primers and probes were adjusted and ranged from 0.16 to 0.4 μM. The plasmid standards, with identical copies/μL, were chosen as the templates. The instrument and program used in this study were consistent with those described previously.

### Analytical sensitivity

We performed multiplex real-time PCR reactions using standard plasmid templates to determine the limit of detection (LOD) of the multiplex detection method. These templates were subjected to 10-fold serial dilution, ranging from 2.183 × 10^5^ to 2.83 × 10^10^ copies/μL for SARS-CoV-2, 1.725 × 10^5^ to 1.725 × 10^10^ copies/μL for CCoV and 0.925 × 10^5^ to 0.925 × 10^10^ copies/μL for FIPV, respectively.

### Analytical specificity

To demonstrate the specificity of the experiment, we evaluated its performance against three target viruses and various other viruses, including FPV, FHV, FCV, CDV, CPV, ICHV, CAV-2, and CPIV. The viral DNA and cDNA templates were previously synthesized and stored in our laboratory prior to use.

### Analytical repeatability

To evaluate the stability of the experiment, we conducted three replicates of the experiment at different times points. For each pathogen, three randomly selected standard plasmids were used with three replicates per reaction. The coefficient of variation (CV) of the Cq values for the samples at each concentration was calculated across the three experiments to assess their repeatability.

### Co-infection simulation and clinical testing experiments

To simulate co-infections, we created combinations of standard samples at various concentrations and maintained consistent ratios of viral mixtures. Two target pathogen plasmid standards were randomly selected at equal concentrations, merged as templates, and subjected to detection using our innovative method. To further replicate co-infection scenarios, one plasmid standard was included at a concentration of 10^7^ copies/μL, while the other was added at a concentration of 10^2^ copies/μL, or equivalent ratios of viral mixtures were employed. Subsequently, we detected the template mixture using our multiplex detection method.

Using a multiplex assay, we tested 48 samples (33 sera samples and 15 ascites samples) obtained from cats, 30 samples obtained from dogs at pet hospitals in China and 3 nasal swabs from individuals exhibiting cold symptoms stored in our laboratory. To evaluate the detection capability, we utilized combinations of three and two viruses (SARS-CoV-2 + CCoV + FIPV, SARS-CoV-2 + CCoV, SARS-CoV-2 + FIPV, CCoV + FIPV), as mock infection samples, similar to the experiments described above. The performance of our established method was evaluated by comparing it with results obtained from classical methods, including the Novel Coronavirus (2019-nCoV) Real Time Multiplex RT-PCR Kit (Shanghai ZJ Bio-Tech Co., Ltd) for SARS-CoV-2, a PCR method according to local standards in Liaoning Province (DB21/T 3093-2018, db-PCR) for CCOV, and EvaGreen real-time PCR established by Guan for FIPV (27). Positive samples identified by both approaches were subsequently sequenced by Shanghai Sangon Biotech Co., Ltd. (Shanghai, China).

## Results

### Plasmid standards preparation

**Figure 1:**
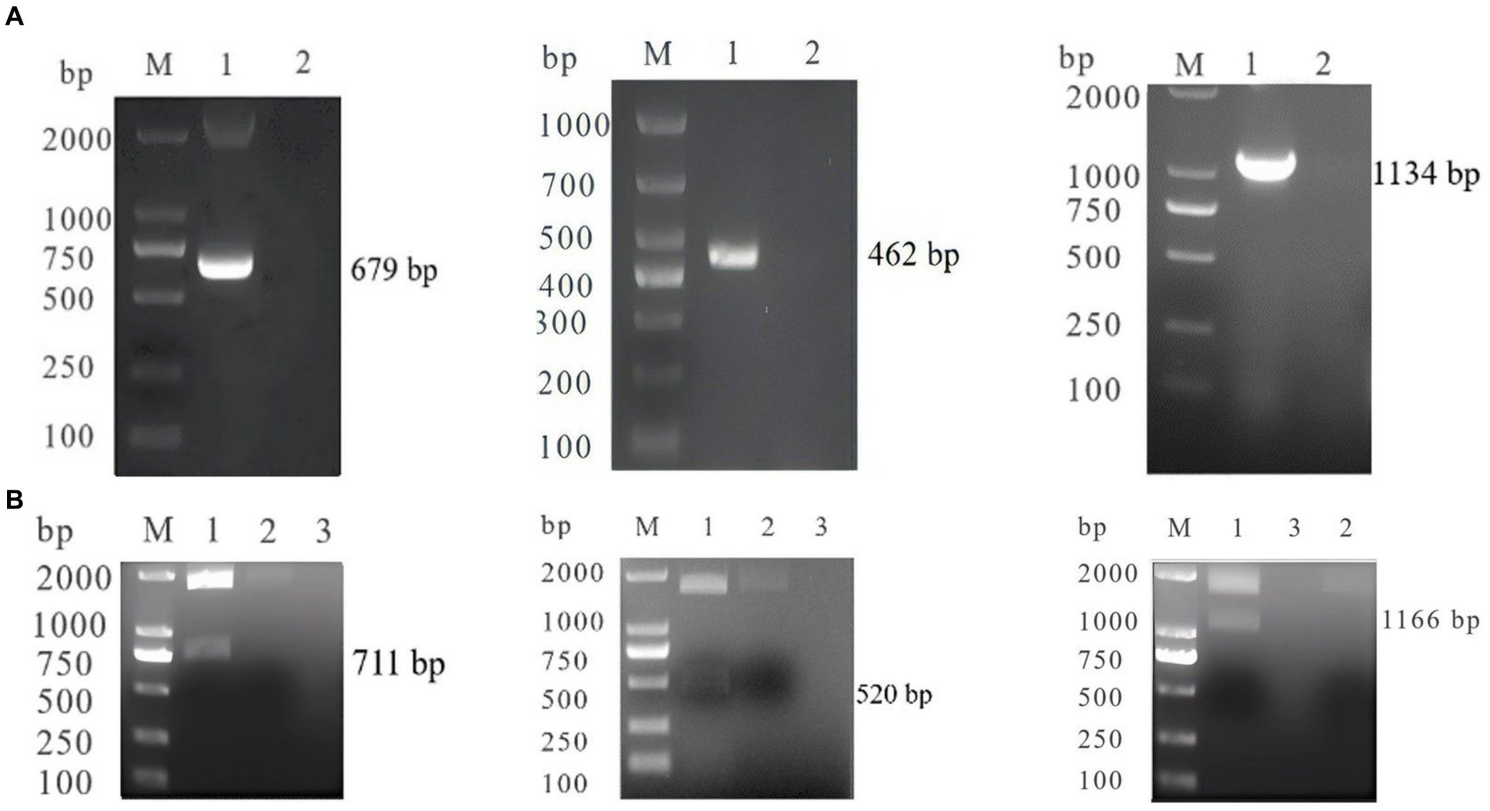
**(A)** PCR identification of SA-N, CC-S, FI-N M: DL200 0/100 0 DNA Marker; 1: SA-N, CC-S, FI-N; 2: Negative control. **(B)** Digestion Identification of SA-N, CC-S, FI-N M: DL200 0/100 0 DNA Marker; 1: SA-N, CC-S, FI-N; 2: empty vector; 3: Negative control.

The products were inserted into the pTOPO-Blunt vector, and subsequent analysis through restriction enzyme digestion and PCR confirmed the successful constructed of SA-N, CC-S, and FI-N (Figure 1).

### System optimization

In the singleplex PCR experiment, we determined the optimal reaction conditions for the primers and probes at different concentrations. For SARS-CoV-2, the optimal concentrations were 0.36 μM for primers and 0.60 μM for probes. For CCoV, the optimal concentrations were 0.48 μM for primers and 0.24 μM for probes. For FIPV, the optimal concentrations were 0.48 μM for primers and 0.36 μM for probes. These concentrations resulted in the lowest Cq values and clear amplification curves.

**Figure 2:**
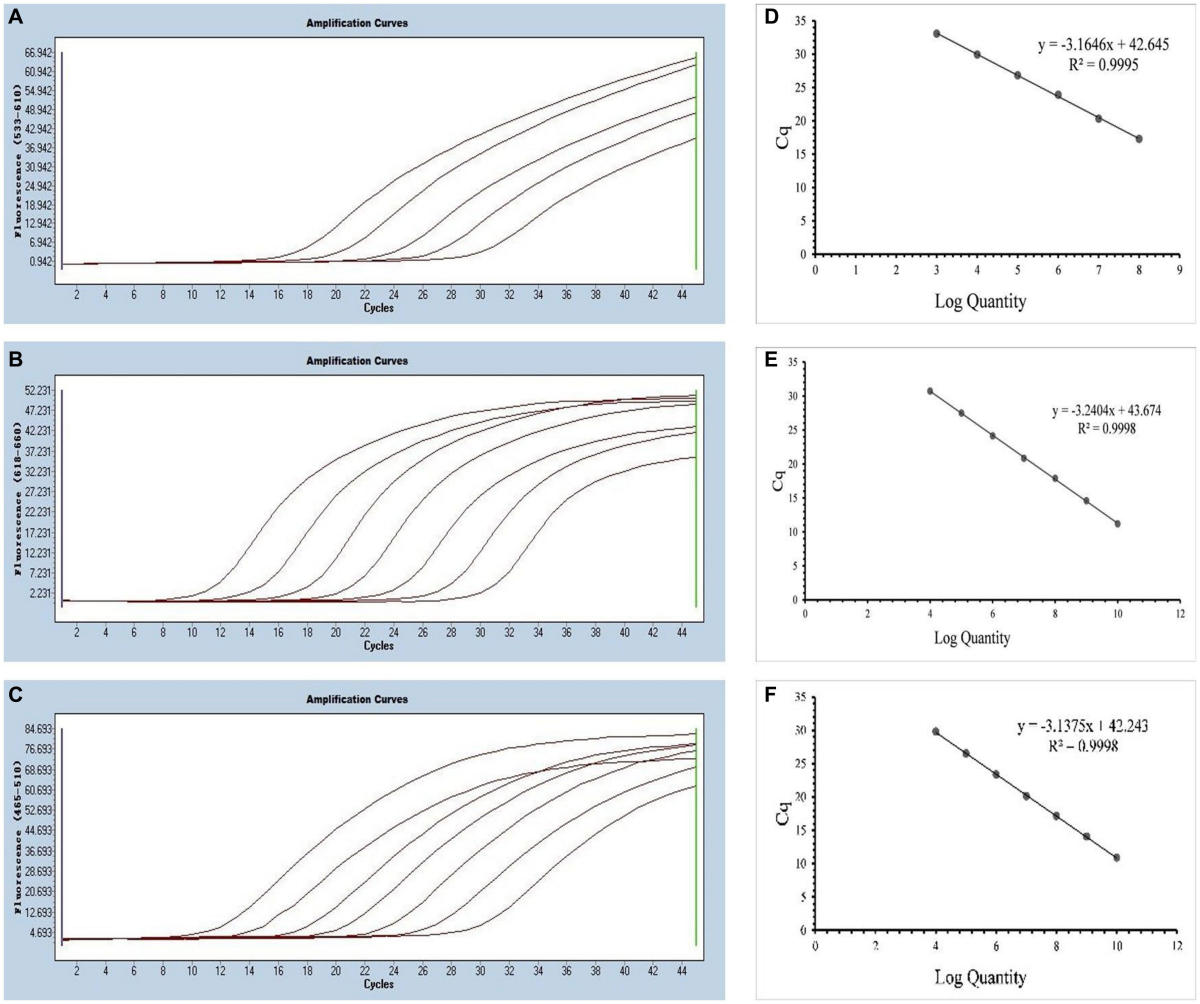
Preparation of plasmid standards. **(A–C)** Amplification curves (X-axis: Cycle, Y-axis: Fluorescence) of SARS-CoV-2, CCoV, and FIPV for each plasmid standard of concentrations with 2.183 × 10^8^copies/μL to 2.183 × 10^4^, 1.725 × 10^10^ to 1.725 × 10^4^ copies/μL, and 0.925 × 10^10^ to 0.925 × 10^4^copies/μL; **(D–F)** Standard curves of plasmid standards of SARS-CoV-2, CCoV, and FIPV. All standard curves were conducted with Microsoft Excel 2016.

The annealing temperature optimization was conducted within a temperature range of 56°C, 58°C, 60°C, and 62°C. The best efficiency was observed at 58°C. For the singleplex real-time PCR, we selected plasmid standards with concentrations ranging from 2.183 × 10^8^ to 2.183 × 10^4^ copies/μL for SARS-CoV-2, 1.725 × 10^10^ to 1.725 × 10^4^ copies/μL for CCoV and 0.925 × 10^10^ to 0.925 × 10^4^ copies/μL for FIPV. The standard curves showed satisfactory amplification efficiency and correlation coefficients: R^2^ = 0.9995 with an E value of 107.00% for SARS-CoV-2; R^2^ = 0.9998 with an E value of 103.51% for CCoV, and R^2^ = 0.9998 with an E value of 108.30% for FIPV (Figure 2). These results confirm the high quality of the plasmid standards and the effectiveness of the primers and probes.

The optimal results were obtained using Texas Red, Cy5, and FAM as reporter dyes, and MGB, BHQ2, and BHQ1 as quencher dyes. It is worth noting that Cy5 has the weakest fluorescence intensity and is highly susceptible to interference from other fluorophores due to its physical properties. Therefore, our primary objective was to enhance the performance of Cy5 fluorophores through individual reaction optimization, ensuring the highest amplification efficiency without compromising the performance of other fluorophores.

**Figure 3:**
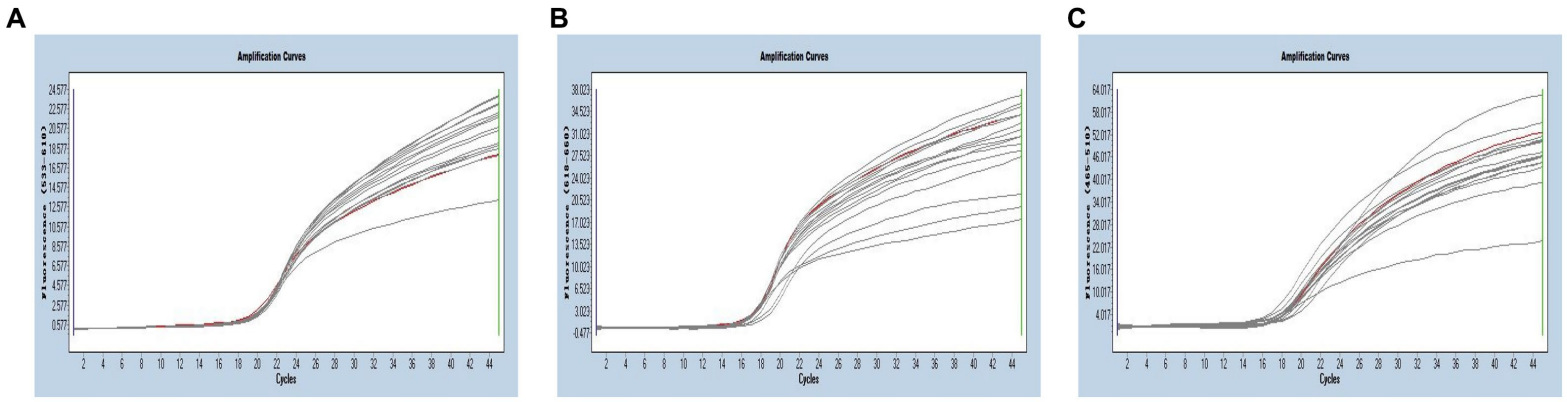
**(A–C)** Amplification curves (X-axis: Cycle, Y-axis: Fluorescence) of SARS-CoV-2, CCoV, and FIPV detected by multiplex real-time PCR with different probe and primer concentrations. The three red lines are the amplification curves of fluorescence of the most suitable reaction tube.

**Figure 4:**
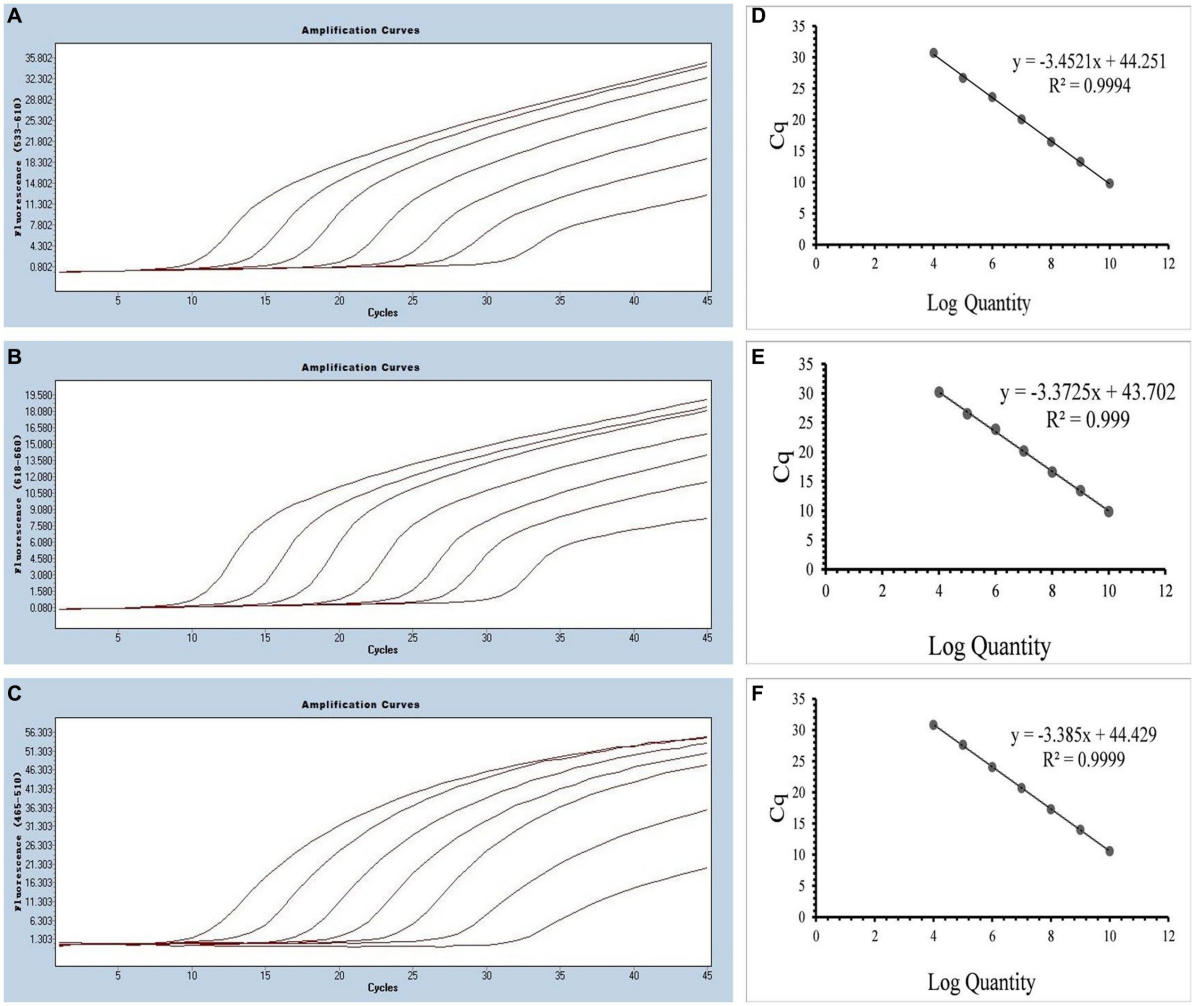
**(A–C)** Amplification curves (X-axis: Cycle, Y-axis: Fluorescence) of SARS-CoV-2, CCoV, and FIPV detected by multiplex real-time PCR for each plasmid standard of concentrations with 2.183 × 10^10^ copies/μL to 2.183 × 10^4^, 1.725 × 10^10^ to 1.725 × 10^4^ copies/μL, and 0.925 × 10^10^ to 0.925 × 10^4^copies/μL; **(D–F)** Standard curves of plasmid standards of SARS-CoV-2, CCoV, and FIPV. All standard curves were conducted with Microsoft Excel 2016.

Multiplex real-time PCR was conducted using primers and probes at varying final concentration ranging from 0.16 to 0.4 μM. The fluorescence intensity and Cq values of all possible combinations were compared, leading to the identification of the optimal final concentrations for primers and probes. Specifically, for SARS-CoV-2 and CCoV, the optimal concentrations were determined to be 0.16 μM for primers and 0.24 μM for probes. For FIPV, both primers and probes were optimized at a concentration of 0.16 μM (Figure 3). To generate the standard curves for the three viruses, plasmids used in the singleplex reactions were employed as templates. The resulting standard curves exhibited excellent linearity, as indicated by the following R^2^ and E values: SARS-CoV-2 R^2^ = 0.9994, E value = 94.85%; CCoV R^2^ = 0.9990, and E value = 97.92%; FIPV R^2^ = 0.9999, E value = 97.42% (Figure 4).

### Analytical sensitivity and specificity

**Figure 5:**
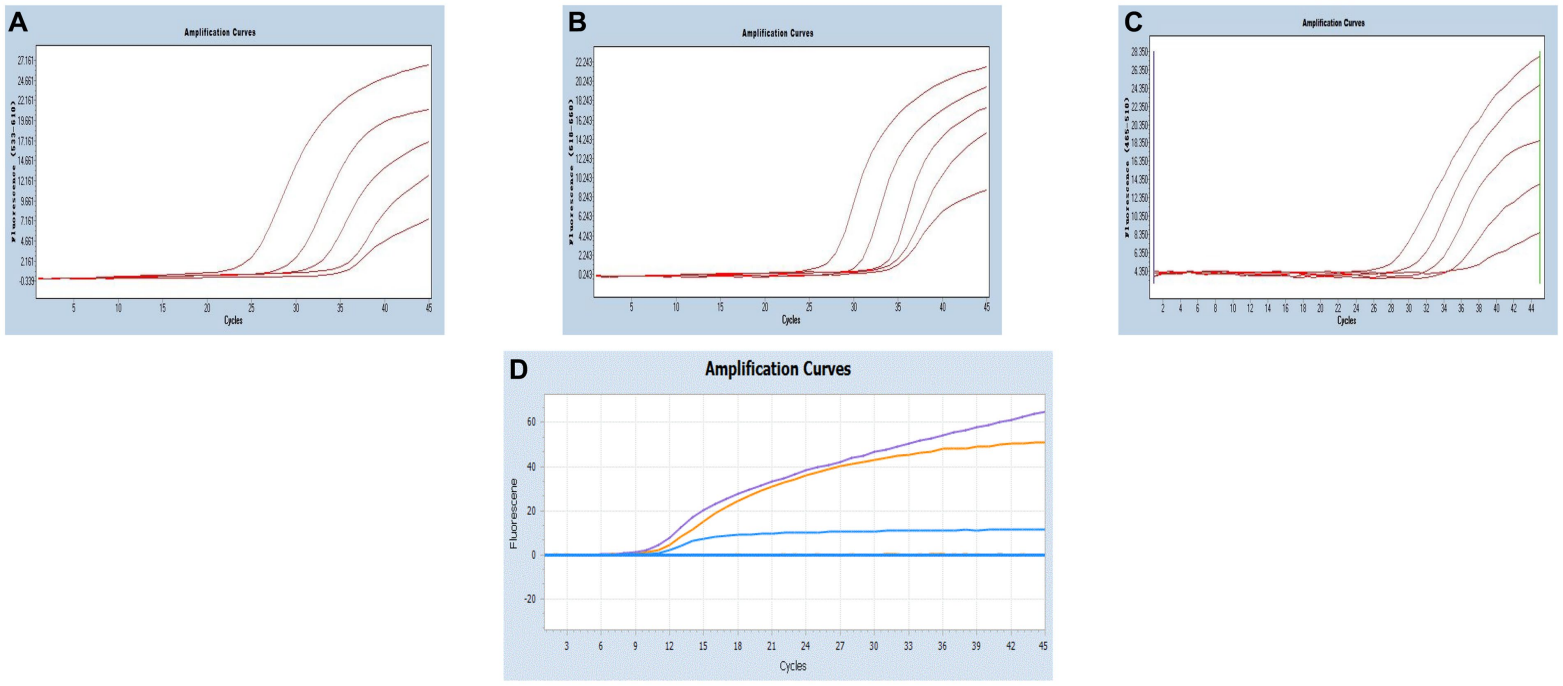
Sensitivity and specificity. **(A–C)** Amplification curves (X-axis: Cycle, Y-axis: Fluorescence) of SARS-CoV-2, CCoV, and FIPV detected by multiplex real-time PCR for each plasmid standard of concentrations with 2.183 × 10^5^copies/μL to 2.183 × 10^1^, 1.725 × 10^5^ to 1.725 × 10^1^ copies/μL, and 0.925 × 10^5^ to 0.925 × 10^1^copies/μL; **(D)** Three amplification curves represent samples positive for SARS-CoV-2 (purple), CCoV (light blue), and FIPV (orange) detected by our multiplex real-time PCR assay; negative samples include FPV, FHV, FCV, CDV, CPV, ICHV, CAV-2, CPIV, and negative control.

By utilizing the optimized system and the plasmid standards, we successfully achieved the LODs for SARS-CoV-2, CCoV and FIPV in both singleplex and multiplex assays. The LODs were determined to be 21.83 copies/μL for SARS-CoV-2, 17.25 copies/μL for CCoV, and 9.25 copies/μL for FIPV, respectively (Figure 5).

Moreover, target viruses have no cross-reactivity with the FPV, FHV, FCV, CDV, CPV, ICHV, CAV-2, and CPIV, indicating a reasonable level of specificity.

### Analytical repeatability

Standard plasmids with concentrations of 10^9^, 10^7^, and 10^5^ copies/μL were chosen to execute three runs in order to measure intra- and inter-assay variation in %CV. As presented in Table 2, the majority of %CV values for the Cq values of the plasmid standard were below 1% (13/18), indicating the high stability of this multiplex detection method.

**Table 2:** Intra- and inter-assay reproducibility of multiplex real-time PCR.

Essay	DNA (copies/μL)	Intra-assay			Inter-assay		
		Mean Cq	SD	CV (%)	Mean Cq	SD	CV (%)
SARS-CoV-2	2.183 × 10^9^	13.25	0.08	0.57%	13.27	0.05	0.35%
	2.183 × 10^7^	20.38	0.09	0.42%	20.07	0.26	1.30%
	2.183 × 10^5^	28.16	0.07	0.26%	28.36	0.23	0.81%
CCoV	1.725 × 10^9^	13.48	0.04	0.28%	13.40	0.08	0.58%
	1.725 × 10^7^	20.51	0.06	0.27%	20.43	0.24	1.18%
	1.725 × 10^5^	27.92	0.08	0.27%	28.32	0.43	1.51%
FIPV	0.925 × 10^9^	13.96	0.07	0.50%	14.16	0.15	1.03%
	0.925 × 10^7^	20.83	0.04	0.17%	20.75	0.04	0.19%
	0.925 × 10^5^	28.79	0.08	0.28%	27.96	0.76	2.72%

### Co-infection simulation and clinical sample detection

**Figure 6:**
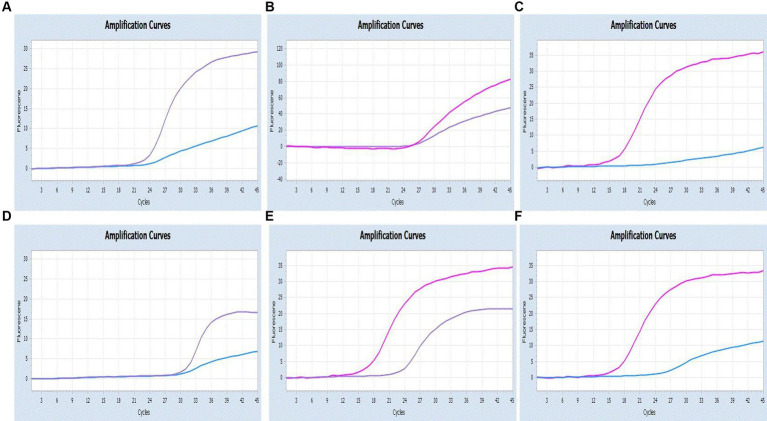
Co-infection simulation experiments with two pathogens (SARS-CoV-2: purple, CCoV: light blue, FIPV: rosy). **(A–C)** Amplification curves (X-axis: Cycle, Y-axis: Fluorescence) of SARS-CoV-2 + CCoV, SARS-CoV-2 + FIPV, and CCoV + FIPV at concentrations of 1 × 10^5^copies/μL; **(D–F)** Amplification curves (X-axis: Cycle, Y-axis: Fluorescence) for viral mixtures of SARS-CoV-2 + CCoV, SARS-CoV-2 + FIPV, and CCoV + FIPV.

**Figure 7:**
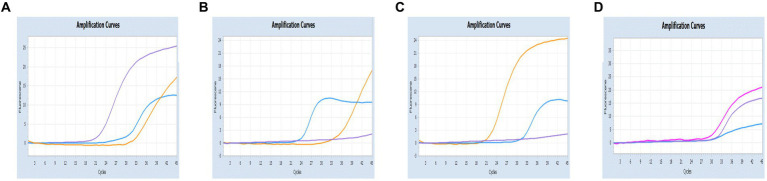
Co-infection simulation experiments with three pathogens (SARS-CoV-2: purple, CCoV: light blue, FIPV: orange or rosy). **(A)** The concentration of plasmid standard of SARS-CoV-2 was 10^7^ copies/μL and the others were 10^2^ copies/μL; **(B)** The concentration of plasmid standard of CCoV was 10^7^ copies/μL and the others were 10^2^ copies/μL; **(C)** The concentration of plasmid standard of FIPV was 10^7^ copies/μL and the others were 10^2^ copies/μL; **(D)** amplification curves (X-axis: Cycle, Y-axis: Fluorescence) for viral mixtures of SARS-CoV-2 + CCoV + FIPV.

As shown in Figures 6, 7, the multiplex assay could detect duplexes or triplexes simulated co-infections of target pathogens, even when present at varying concentrations.

As showed in Table 3, we tested a total of 33 sera samples and 15 ascites samples from cats, 30 sera samples from dogs, 3 nasal swabs from human and four viral mixtures. To assess the agreement between each pair of diagnostic techniques for the same case, kappa validation was performed. Results indicate a high level of consistency between the outcomes of the two diagnostic techniques (SARS-CoV-2: Kappa = 1, *p* = 0.014^*^; CCoV: Kappa = 0.921, *p* = 0.000^**^; FIPV: Kappa = 0.882, *p* = 0.000^**^).

**Table 3:** The positivity rate of multiplex real-time and RT-PCR tests for 85 samples.

Clinical Samples	Multiplex Real-Time PCR Assay Results			The Proven Method		
	Positive number	Negative number	Detected rate	Positive number	Negative number	Detected rate
SARS-CoV-2	4	2	66.67(4/6)	4	2	66.67(1/6)
CCoV	9	24	27.27(9/33)	8	25	24.24(8/33)
FIPV	26	25	50.98(26/51)	25	26	49.02(25/51)

## Discussion

To the best of our knowledge, this study represents the first multiplex real-time PCR assay capable of simultaneously detecting SARS-CoV-2, CCoV, and FIPV. Among these pathogens, SARS-CoV-2, which causes coronavirus disease 2019 (COVID-19), has a high mortality rate and spreads rapidly, impacting global human health significantly (28). Although widespread, CCoV is not regarded as a highly lethal canine intestinal virus that has not caused substantial economic losses (29, 30). In contrast, FIPV is associated with lower prevalence but high mortality, and there are currently no approved treatments for it in veterinary practice (31). The lack of rapid and simple detection methods for disease surveillance hinders efficient control and eradication efforts. Therefore, this study provides a valuable tool for assessing the prevalence and geographic distribution of suspected and co-infected animals. Additionally, it facilitates investigations into coronavirus epidemiology, thereby contributing to advancements in both animal and public health.

Cross-species transmission is a major concern, as coronaviruses possess the ability to adapt to new hosts, posing serious threats to both human and animal health (32). The increasing proximity between humans and dogs/cats exacerbates the risk of virus transmission to humans (33). Furthermore, SARS-CoV-2 has been detected in various animals, and coronavirus has also been identified in human beings (34). Vlasova et al. (33) firstly isolated CCoV RNA from human pneumonia patients in Sarawak, Malaysia, between 2017 and 2018, identifying the virus as a novel canine-feline recombinant coronavirus named CCoV-HuPn-2018. Lednicky et al. (35) reported the isolation of a novel recombinant canine coronavirus from visitors to Haiti, resembling the Malaysian virus found by Vlasova et al. (35). In a study conducted in Arkansas, United States, in 2010, three isolates with high homology to FCoV were detected in patients with acute influenza-like (36). While it has been demonstrated that cats can be infected with CCoV under experimental conditions, it remains uncertain whether CCoV and FCoV readily cross the species barrier (25, 37–39). These examples highlight the potential for animals to serve as a reservoir for the emergence of novel recombinant coronaviruses, thereby expanding their host tropism to humans. Therefore, the development of an efficient and accurate detection method, such as multiplex real-time PCR, for identifying and monitoring co-infections is imperative. These findings underscore the threat of animal coronaviruses to public health. Given the seriousness of the current pandemic and the potential impact of animal hosts on the transmission dynamics of SARS-CoV-2 in the human population, it is crucial to establish effective surveillance systems to monitor animal coronavirus infections.

The clinical diagnosis of coronavirus presents challenges, and laboratory diagnostic methods are indispensable. FECV and FIPV are unable to differentiate between serotypes, posing challenges in accurately identifying antibodies. While immunohistochemistry is often considered the gold standard for diagnosis, its operation is complex. In contrast, real-time PCR has emerged as an efficient, sensitive, specific, and quantitative technique for detecting viral load, gaining prevalence in clinical practice. Recent studies have reported detection methods focusing on single viruses that outperform conventional PCR (40). For SARS-CoV-2, real-time PCR is widely considered the gold standard, with numerous diagnostic tests available on the market targeting primarily the ORF1ab, N, E genes (41, 42). Lu et al. (43) developed a diagnostic panel consisting of three real-time reverse transcription PCR assays targeting the N gene, with a detection limit of 5 copies/reaction of quantified RNA transcripts. Additionally, Daniel et al. developed two 1-step quantitative real-time reverse-transcription PCR assays to detect ORF1b and N of the viral genome with detection limits below 10 copies per reaction (44). As early as 2004, Decaro et al. established a real-time PCR assay for CCoV against ORF5 (M gene) with a detection limit of 10 copies of CCoV standard RNA. Recently, Dema et al. used this method to investigate viral pathogens associated with canine gastroenteritis (45, 46). And Felten et al. (47) designed hydrolysis probes to detect cat cerebrospinal fluid using 7b-real-time PCR. While multiplex PCR lacks the sensitivity advantage of multiplex real-time PCR, singleplex real-time PCR assays are inconvenient for detecting co-infection with multiple pathogens simultaneously. Furthermore, multiplex real-time PCR offers improved detection capability and lower laboratory costs in less time. Wang et al. (48) developed a multiplex real-time PCR assay capable of differentially diagnosing four viruses responsible for canine diarrhea, including CCoV, with 100-fold higher sensitivity than other multiplex PCR. Sun et al. (49) developed a duplex real-time PCR assay based on SYBR Green I for FPV and FCoV. However, the dye method exhibits poorer specificity compared to the probe assay, and the presence of primer dimers or non-specific products significantly impact the reaction. Additionally, SYBR Green I may inhibit the reaction (50).

Interference from selected fluorescence channels has been corrected through color compensation, following the provided instructions (51, 52). The utilization of S and N gene sequences verified the primer conservation and probe specificity. However, given the rapid evolution and variable nature of coronaviruses, periodic verification of primer and probe sequences may be necessary. The ability to accurately detect viruses at lower concentrations facilitates early diagnosis and prevention, thereby endowing our assay with robust surveillance capabilities. Nevertheless, heightened sensitivity also increases the risk of false positive results, necessitating the implementation of more stringent measures to prevent nucleic acid contamination.

The assay exhibits excellent analytical and clinical performance, showcasing its high efficiency, sensitivity and specificity. Comparability to the gold standard assay. During clinical testing, our method demonstrated higher sensitivity or consistency compared to validated methods. It achieved simultaneous detection of three target viruses in a single sample. However, it is pity that the sample used was artificially mixed viral fluid rather than samples obtained under natural conditions. Importantly, our developed method enables the simultaneous detection of multiple pathogens in a single reaction, providing a more convenient approach to identify co-infections and significantly reduce labor and material costs.

## Conclusion

This report presents the development of an innovative real-time PCR technique capable of simultaneously detecting SARS-CoV-2, CCoV and FIPV with high accuracy. This method offers a more suitable approach for large-scale diagnosis and prevalence investigations. Notably, this technique not only saves considerable time and laboratory resources, but also provides rapid results, high sensitivity, specificity and excellent reproducibility, which renders it an ideal choice for diagnostic laboratories. Moreover, the simultaneous testing capability enhances detection capacity while reducing workload and cost burden.

## Data availability statement

The datasets presented in this study can be found in online repositories. The names of the repository/repositories and accession number(s) can be found in the article/Supplementary material.

## Author contributions

YL: Methodology, Writing – original draft. ZZ: Software, Writing – review & editing. JD: Project administration, Writing – review & editing. XZ: Writing – review & editing. CP: Visualization, Writing – review & editing. CY: Funding acquisition, Writing – review & editing. WS: Supervision, Writing – review & editing.
