# Supplementary material for: Development of multiplex real-time PCR for simultaneous detection of SARS-CoV-2, CCoV, and FIPV
Source: Front Vet Sci. 2024 Jul 10;11:1337690. doi: 10.3389/fvets.2024.1337690 (PMC11266814; doi:10.3389/fvets.2024.1337690)
Supplement: Supplementary file 1 [file Table_1.pdf]

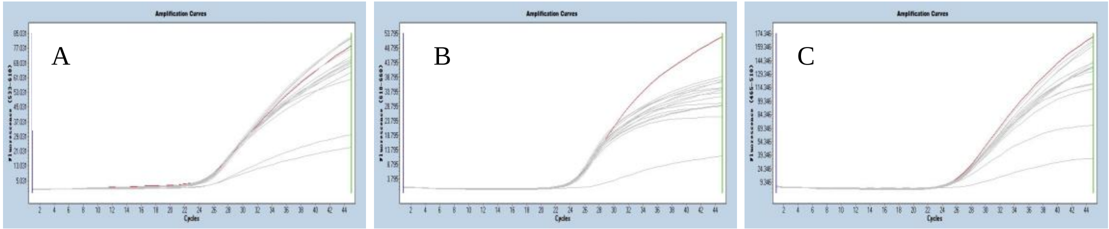

Figure 1. A-C: amplification curves (X-axis: Cycle, Y-axis: Fluorescence) of SARS-CoV-2, CCoV, and FIPV detected by singleplex real-time PCR with different probe and primer concentrations. The three red lines are the amplification curves of fluorescence of the most suitable reaction tube.

Table 1. Data of standard curve for singleplex real-time PCR

10-fold gradient dilution	SARS-CoV-2		CCoV		FIPV	
	Concentration(copies/μL)	Cq	Concentration(copies/μL)	Cq	Concentration(copies/μL)	Cq
10^{10}			1.725×10^{10}	11.20	0.925×10^{10}	10.91
10^{9}			1.725×10^{9}	14.59	0.925×10^{9}	14.07
10^{8}	2.183×10^{8}	17.30	1.725×10^{8}	17.90	0.925×10^{8}	17.15
10^{7}	2.183×10^{7}	20.34	1.725×10^{7}	20.84	0.925×10^{7}	20.12
10^{6}	2.183×10^{6}	23.91	1.725×10^{6}	24.16	0.925×10^{6}	23.36
10^{5}	2.183×10^{5}	26.84	1.725×10^{5}	27.50	0.925×10^{5}	26.54
10^{4}	2.183×10^{4}	29.95	1.725×10^{4}	30.75	0.925×10^{4}	29.81
10^{3}	2.183×10^{3}	33.10				

Table 2. Data of standard curve for multiplex real-time PCR

10-fold gradient dilution	SARS-CoV-2		CCoV		FIPV	
	Concentration(copies/μL)	Cq	Concentration(copies/μL)	Cq	Concentration(copies/μL)	Cq
10^{10}	2.183×10^{10}	9.81	1.725×10^{10}	9.86	0.925×10^{10}	10.59
10^{9}	2.183×10^{9}	13.26	1.725×10^{9}	13.44	0.925×10^{9}	14.00
10^{8}	2.183×10^{8}	16.46	1.725×10^{8}	16.61	0.925×10^{8}	17.30
10^{7}	2.183×10^{7}	20.06	1.725×10^{7}	20.15	0.925×10^{7}	20.71
10^{6}	2.183×10^{6}	23.63	1.725×10^{6}	23.89	0.925×10^{6}	24.07
10^{5}	2.183×10^{5}	26.70	1.725×10^{5}	26.52	0.925×10^{5}	27.63
10^{4}	2.183×10^{4}	30.68	1.725×10^{4}	30.19	0.925×10^{4}	30.84

Table 3. Data of sensitivity test of multiplex for multiplex real-time PCR

10-fold gradient dilution	SARS-CoV-2		CCoV		FIPV	
	Concentration(copies/μL)	Cq	Concentration(copies/μL)	Cq	Concentration(copies/μL)	Cq
10^{5}	2.183×10^{5}	26.13	1.725×10^{5}	28.20	0.925×10^{5}	27.74
10^{4}	2.183×10^{4}	31.02	1.725×10^{4}	31.57	0.925×10^{4}	30.62
10^{3}	2.183×10^{3}	34.60	1.725×10^{3}	34.93	0.925×10^{3}	33.23
10^{2}	2.183×10^{2}	37.87	1.725×10^{2}	35.99	0.925×10^{2}	35.60
10^{1}	2.183×10^{1}	39.82	1.725×10^{1}	36.89	0.925×10^{1}	37.86

Table 4. Data for multiplex real-time PCR detecting the clinical samples.

Samples	Source	Types	SARS-CoV-2		CCoV		FIPV	
			Commercial kits	multiplex real-time PCR	Db-PCR	multiplex real-time PCR	EvaGreen real-time PCR	multiplex real-time PCR
1	cat	Sera	–	–	–	–	–	–
2	cat	Sera	–	–	–	–	–	–
3	cat	Sera	–	–	–	–	–	–
4	cat	Sera	–	–	–	–	–	–
5	cat	Sera	–	–	–	–	–	–
6	cat	Sera	–	–	–	–	–	–
7	cat	Sera	–	–	–	–	–	–
8	cat	Sera	–	–	–	–	–	–
9	cat	Sera	–	–	–	–	–	–
10	cat	Sera	–	–	–	–	–	–
11	cat	Sera	–	–	–	–	–	–

12	cat	Sera	−	−	−	−	−	−
13	cat	Sera	−	−	−	−	−	−
14	cat	Sera	−	−	−	−	−	−
15	dog	Sera	−	−	−	−	−	−
16	dog	Sera	−	−	−	−	−	−
17	dog	Sera	−	−	−	−	−	−
18	dog	Sera	−	−	−	−	−	−
19	dog	Sera	−	−	−	−	−	−
20	dog	Sera	−	−	−	−	−	−
21	dog	Sera	−	−	+	+	−	−
22	dog	Sera	−	−	−	−	−	−
23	dog	Sera	−	−	−	−	−	−
24	dog	Sera	−	−	+	+	−	−
25	cat	Sera	−	−	−	−	−	−
26	cat	Sera	−	−	−	−	−	−
27	cat	Sera	−	−	−	−	−	−
28	cat	Sera	−	−	−	−	+	−
29	cat	Sera	−	−	−	−	−	−
30	cat	Sera	−	−	−	−	−	−
31	cat	Sera	−	−	−	−	−	−
32	dog	Sera	−	−	−	−	−	−
33	dog	Sera	−	−	−	−	−	−
34	dog	Sera	−	−	−	−	−	−
35	dog	Sera	−	−	−	−	−	−
36	dog	Sera	−	−	−	−	−	−
37	dog	Sera	−	−	−	−	−	−
38	dog	Sera	−	−	−	−	−	−
39	human	nasal swabs	−	−	−	−	−	−
40	human	nasal swabs	+	+	−	−	−	−
41	dog	Sera	−	−	−	−	−	−
42	dog	Sera	−	−	−	−	−	−
43	dog	Sera	−	−	−	−	−	−
44	dog	Sera	−	−	−	−	−	−
45	dog	Sera	−	−	−	−	−	−
46	dog	Sera	−	−	−	−	−	−
47	dog	Sera	−	−	+	+	−	−
48	dog	Sera	−	−	−	+	−	−
49	dog	Sera	−	−	−	−	−	−
50	dog	Sera	−	−	−	−	−	−
51	dog	Sera	−	−	−	−	−	−
52	dog	Sera	−	−	+	+	−	−
53	dog	Sera	−	−	+	+	−	−
54	cat	Sera	−	−	−	−	−	−
55	cat	Sera	−	−	−	−	−	+
56	cat	Sera	−	−	−	−	+	+
57	cat	Sera	−	−	−	−	+	+
58	cat	Sera	−	−	−	−	+	+
59	human	nasal swabs	−	−	−	−	−	−
60	cat	Sera	−	−	+	−	+	+
61	cat	Sera	−	−	−	−	−	+
62	cat	Sera	−	−	−	−	+	+

63	cat	Sera	−	−	−	−	+	+
64	cat	Sera	−	−	−	−	+	+
65	cat	Sera	−	−	−	−	+	+
66	cat	Sera	−	−	−	−	+	+
67	cat	ascites	−	−	−	−	−	−
68	cat	ascites	−	−	+	−	+	+
69	cat	ascites	−	−	−	−	+	+
70	cat	ascites	−	−	−	−	−	−
71	cat	ascites	−	−	−	−	+	+
72	cat	ascites	−	−	−	−	+	+
73	cat	ascites	−	−	−	−	+	+
74	mixture	SARS-CoV-2 + CCoV+ FIPV，	+	+	+	+	+	+
75	cat	ascites	−	−	−	−	+	+
76	cat	ascites	−	−	−	−	+	+
77	cat	ascites	−	−	−	−	+	+
78	cat	ascites	−	−	−	−	+	+
79	cat	ascites	−	−	−	−	+	+
80	cat	ascites	−	−	−	−	−	−
81	cat	ascites	−	−	−	−	+	+
82	cat	ascites	−	−	−	−	+	+
83	mixture	SARS-CoV-2 + CCoV	+	+	+	+	−	−
84	mixture	SARS-CoV-2 + FIPV	+	+	−	−	+	+
85	mixture	CCoV+ FIPV	−	−	+	+	+	+

"+" indicates positive; "−" indicates negative
